# Small Molecule Inhibited Parathyroid Hormone Mediated cAMP Response by N–Terminal Peptide Binding

**DOI:** 10.1038/srep22533

**Published:** 2016-03-02

**Authors:** Amit Kumar, Monika Baumann, Jochen Balbach

**Affiliations:** 1 Astbury Centre for Structural Molecular Biology, School of Molecular and Cellular Biology, University of Leeds, Leeds LS2 9JT, UK; 2 Institute of Physics, Biophysics, Martin–Luther–University Halle–Wittenberg, Germany; 3 Centre for Structure und Dynamics of Proteins (MZP), Martin–Luther–University Halle–Wittenberg, Germany

## Abstract

Ligand binding to certain classes of G protein coupled receptors (GPCRs) stimulates the rapid synthesis of cAMP through G protein. Human parathyroid hormone (PTH), a member of class B GPCRs, binds to its receptor *via* its N–terminal domain, thereby activating the pathway to this secondary messenger inside cells. Presently, GPCRs are the target of many pharmaceuticals however, these drugs target only a small fraction of structurally known GPCRs (about 10%). Coordination complexes are gaining interest due to their wide applications in the medicinal field. In the present studies we explored the potential of a coordination complex of Zn(II) and anthracenyl–terpyridine as a modulator of the parathyroid hormone response. Preferential interactions at the N–terminal domain of the peptide hormone were manifested by suppressed cAMP generation inside the cells. These observations contribute a regulatory component to the current GPCR–cAMP paradigm, where not the receptor itself, but the activating hormone is a target. To our knowledge, this is the first report about a coordination complex modulating GPCR activity at the level of deactivating its agonist. Developing such molecules might help in the control of pathogenic PTH function such as hyperparathyroidism, where control of excess hormonal activity is essentially required.

Usage of mixed–ligand complexes is a rapidly growing area due to their applications in the industrial, environmental and medicinal fields^1^. Their applications range from anticancer^2^,^3^,^4^,^5^ to antimicrobial^3^,^4^,^6^,^7^ and antifungal activities^3^,^4^,^8^. For the normal function of biochemical pathways in organisms transition metal ions are essential components. Therefore, it is not surprising that coordination complexes are of great interest. These complexes exhibit versatile spectral and electrochemical properties with tunable coordination chemistry, which subsequently offer an enormous scope for the design of new species. Complexes of various metal ions are under intense investigation to highlight their potential in medicinal chemistry^9^,^10^,^11^. Zn is one of the most common metal ions found in biological systems^12^,^13^, e.g. playing an important role in many transcription factors. Diverse biological functions of Zn(II) result from its redox stability and variability in coordination chemistry. Zn based coordination compounds are gaining interest because they show better biological responses towards infectious organisms than free metals^2^,^5^,^6^,^7^,^8^. For instance, Zn complexes have been studied to induce phosphorylation of the Akt downstream effector glycogen synthase kinase 3β and attributed useful tools for regulating glucose metabolism or serve as lead structures for developing antidiabetic drugs^14^. Other studies showed the inhibition of caspase-3 activity and promotion of ErbB1-ErbB2 heterodimerization by Zinc pyrithione^15^ and inhibition of cyclin-dependent kinase CDK1^16^. Other examples include Zn complexes exhibiting the antibacterial/antimicrobial, anticancer activities, interacting with the DNA and inducing protein aggregation^2^,^5^,^6^,^7^,^8^,^17^.

At present, cell surface receptors including GPCRs have been frequently targeted by potential drug molecules for pharmacological applications^18^,^19^,^20^,^21^. GPCRs form the largest family of human transmembrane proteins and play a major role in various physiological functions, including cell–cell communication, cell differentiation, metabolism and synaptic transmission. Various signaling molecules including hormones, neurotransmitters, chemokines, ions, tastants and odorants bind to GPCRs^22^ and activate the pathways to secondary messengers such as inositol trisphosphate, diacylglycerol, cGMP or cAMP^23^,^24^,^25^,^26^. Agonists such as PTH, PTH related peptide or tuberoinfundibular peptide of 39 residues (TIP39) interact with parathyroid hormone receptors 1 and 2 (PTH1R/2R), both members of class B GPCRs, and activate intracellular signaling, in turn modulating cellular function, including the skeletal, endocrine, cardiovascular and nervous systems^24^,^27^,^28^,^29^,^30^. Therefore, PTH(1–34) and PTH(1–84) are also used as drugs against osteoporosis^31^,^32^.

PTH is secreted by the parathyroid gland as an 84 residue peptide and regulates the calcium and phosphate levels in the blood stream. However, in the case of hyperparathyroidism, PTH level become elevated which triggers the excess release of calcium into the bloodstream. This calcium is taken from the bones, which subsequently leds to bone fatigue. The elevated levels of calcium may increase in the urine and cause kidney stones. At the molecular level, basic signal transduction starts when PTH binds to its receptors leading to their activation and subsequent generation of secondary messengers^22^,^24^,^33^. PTH binding and receptor activation is carried out by the N–terminal part of the peptide^34^. It implies that one possible regulation of hormonal activity could be at the ligand level, particularly the N–terminal domain of PTH. Recently, it has been reported that N–terminal phosphorylation at Ser1, Ser3 and Ser17 of PTH abolished receptor activity^24^.

As GPCRs are involved in many diseases, they are targets for approximately 40% of all human modern medicinal drugs^35^,^36^. However, these drugs target only a small fraction (about 10%) of known GPCRs^37^. Within the context of receptors including GPCRs, typically available drug molecules for pharmacological applications directly target the receptors^18^,^19^,^20^,^21^. However, literature is limited with regards to molecules which bind to the agonist to modify its receptor activating function. A potential class of molecules for this purpose could be organic/coordination/mixed–ligand complexes. In mixed–ligand complexes one metal ion can replace another metal ion bearing the original skeletal system. A metal replacement can lead to suppressed or negligible biological responses^9^. Previously we have developed the Cu(II) complex of anthracenyl terpyridine which showed nuclease and antiprolifirative activity for a broad range of cancer cells^9^. In principle, Zn(II) is less toxic than Cu(II) and it is redox silent therefore, this metal was chosen in exchange of Cu(II). Moreover, metal insertion is essential as anthracenyl terpyridine is water insoluble in the absence of the metal. A replacement of Zn(II) in anthracenyl terpyridine did not show nuclease or antiprolifirative activities^9^.

In the present studies, a mixed–ligand of Zn(II) and anthracenyl terpyridine (Fig. 1)^9^ has been employed as a modulator of the PTH response by directly targeting the agonist. We found that the Zn complex binds PTH with micromolar affinity without inducing major conformational changes of the peptide hormone. NMR spectroscopy revealed that the Zn complex preferentially binds to the N–terminal domain of PTH which is important for receptor binding and activation. We attribute specific hydrophobic interactions between PTH and the Zn complex for binding. An intracellular signalling assay revealed that interactions at the N–terminal domain led to suppressed secondary messenger cAMP generation in cultured cell. Thus a mixed–ligand metal complex provides an additional regulatory component for specific agonist inhibition of GPCR activation.

## Results

### Binding revealed by fluorescence spectroscopy

The natural form of parathyroid hormone comprises 84 residues, however, PTH(1–34) is sufficient for receptor activation^38^. PTH contains one tryptophan residue at position 23. Thus it can be used as a highly sensitive fluorescent probe for the analysis of ligand binding by fluorescence spectroscopy. PTH is composed of structured sections mainly from positions 1–37, whereas the C–terminal part is largely unstructured^39^,^40^. When excited at 280 nm, PTH showed the intrinsic fluorescence emission maxima centered at 348 nm, which is characteristic of Trp exposed to the solvent, as supported by the NMR structure. In order to establish the binding a titration experiment was carried out where the PTH(1–34) concentration was kept constant and the concentration of the Zn complex was varied. A non–linear decrease in fluorescence intensity was observed with increasing concentration of the Zn complex suggesting quenching of the tryptophan fluorescence upon binding of the Zn complex.

**Figure 2:**
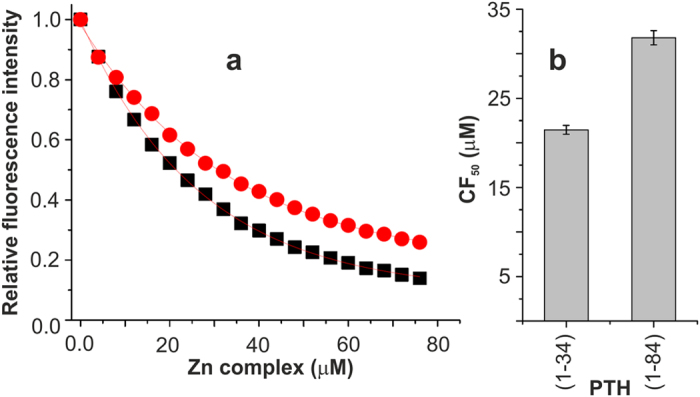
Fluorescence quenching of the PTH peptide hormone by the Zn complex. (**a**) Relative fluorescence intensity plot and best fit (red lines) according to equation for ■ – PTH(1–34) and 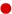 – PTH(1–84). (**b**) CF_50_ in μM (concentrations of the Zn complex required for 50% fluorescence quenching) plot.

The fluorescence quenching behavior was analyzed according to the equation given in the experimental section. The data showed the exponential decay as a function of Zn complex (Fig. 2a and Figure S1). Data fitting resulted in a *K*_D_ value as 27 ± 1.8 μM indicating a moderate binding affinity of the Zn complex to PTH(1–34) and a stoichiometry of 1.9 (numbers of binding sites with equal affinities). In order to evaluate the interaction at the C–terminal part of the PTH and its role in binding, full length PTH(1–84) was taken and its binding was established. Similar to the PTH(1–34), PTH(1–84) also showed the quenching in fluorescence intensity as a function of the Zn complex concentration. This resulted in a *K*_D_ value of 34 ± 1.7 μM and a stoichiometry of 2.0. Thus, the Zn complex showed almost the same affinity towards PTH(1–34) and PTH(1–84) and both peptide hormones interact with two molecules of Zn complex. Zn–perchlorate up to 200 μM did not show any significant change in fluorescence intensity (Figure S1). In a second control experiment, 9-anthracenemethanol was titrated against PTH(1–84) which showed a significantly weak affinity (*K*_D_ = 3.52 ± 0.38 mM) compared to the Zn complex (Figure S2). This highlights the crucial role of the organic skeletal together with metal ion for efficient binding beyond an unspecific hydrophobic interaction.

A closer analysis of the fluorescence data suggested that there is negligible difference in the quenching behavior of PTH(1–34) and PTH(1–84). Data were further analyzed based upon the relative intensity plot (Fig. 2a). The plot of PTH(1–84) suggests ~75% reduced intensity at the end of the titration (76 μM of Zn complex), it was ~86% in the case of PTH(1–34) at the same concentration. This behavior can be further understood from the concentrations of the Zn complex required for 50% quenching of fluorescence intensity (CF_50_)^41^. The CF_50_ for PTH(1–34) was 21.46 ± 0.5 μM and for PTH(1–84) it was 31.8 ± 0.8 μM (Fig. 2b). These analyses confirm that the Zn complex binds to PTH(1–34) and PTH(1–84) in a similar way, although there are trivial differences in the interactions.

### Binding revealed by CD spectroscopy

**Figure 3:**
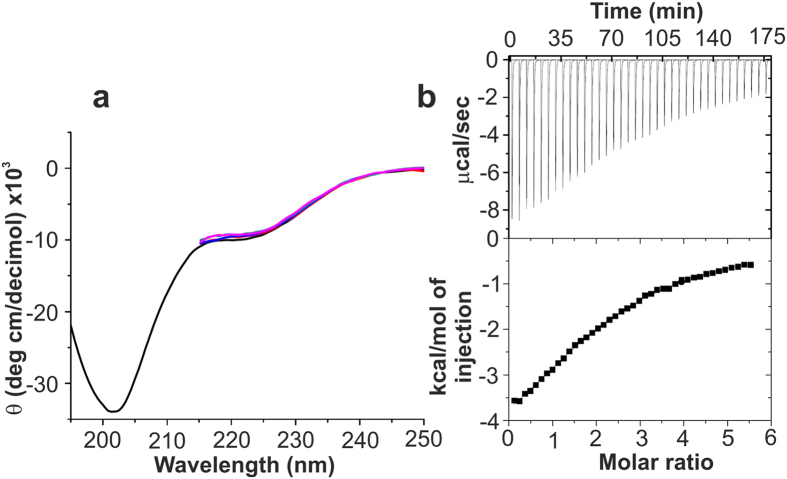
Binding analysis of the Zn complex with peptide hormones. (**a**) CD spectra of PTH(1–34) upon titration with the Zn complex (10 μM to 50 μM). The black spectrum corresponds to free PTH(1–34). Spectra beyond 215 nm could not be recorded due to the high absorption upon addition of the Zn complex (**b**) ITC profile and best fit of the interaction of Zn complex with PTH(1–34). Top: raw data obtained from injections of the Zn complex into the PTH(1–34) solution. Bottom represents the integrated curve showing the experimental points (■) and best fit (–).

Possible conformational changes of peptide secondary structure induced in PTH(1–34) or PTH(1–84) upon binding the Zn complex can be revealed by circular dichroism (CD) spectroscopy. The CD spectrum of PTH(1–34)^42^ and PTH(1–84)^43^ showed minima at 222 nm, characteristic of α–helical content. A pronounced minimum at 202 nm is attributed towards unstructured regions at the C – terminus of the peptide which is dominating the far UV CD spectrum. The binding of the Zn complex to the PTH(1–34) or PTH(1–84) did not show significant changes in the CD spectra and thus no secondary structural changes of the peptides (Fig. 3a and Figure S3).

### Thermodynamics of binding by ITC studies

ITC is a well suited method to evaluate protein-ligand interaction^41^,^44^. The interaction of the Zn complex with PTH(1–34) and PTH(1–84) was evaluated by ITC at 25 °C. Zn complex binding was exothermic in nature for both the peptides (Fig. 3b and Figure S4). Data fitting gave n values of 2.28 ± 0.13 and 1.93 ± 0.04 for PTH(1–34) and PTH(1–84), respectively. This is suggestive of at least two molecules of the Zn complex interacting with the peptides. The isotherm yields the following thermodynamic parameters: (*K*_D_ = 55.06 ± 0.06 μM, ∆H = −5.9 ± 0.1 kcal mol^−1^and ∆S = −5.37 cal mol^−1^K^−1^) and (*K*_D_ = 54.1 ± 0.08 μM, ∆H −11.25 ± 0.3 kcal mol^−1^ and ∆S = −7.15 cal mol^−1^K^−1^) for PTH(1–34) and PTH(1–84), respectively. The dissociation constant in the low micromolar range and negative ∆H indicates that the Zn complex interacts with the peptide hormones *via* rather weak interactions. The enthalpic gain counterbalances the unfavorable loss in entropy of the system upon complex binding.

### NMR titration of Zn complex with ^15^N–PTH(1–34) and ^15^N–PTH(1–84)

**Figure 4:**
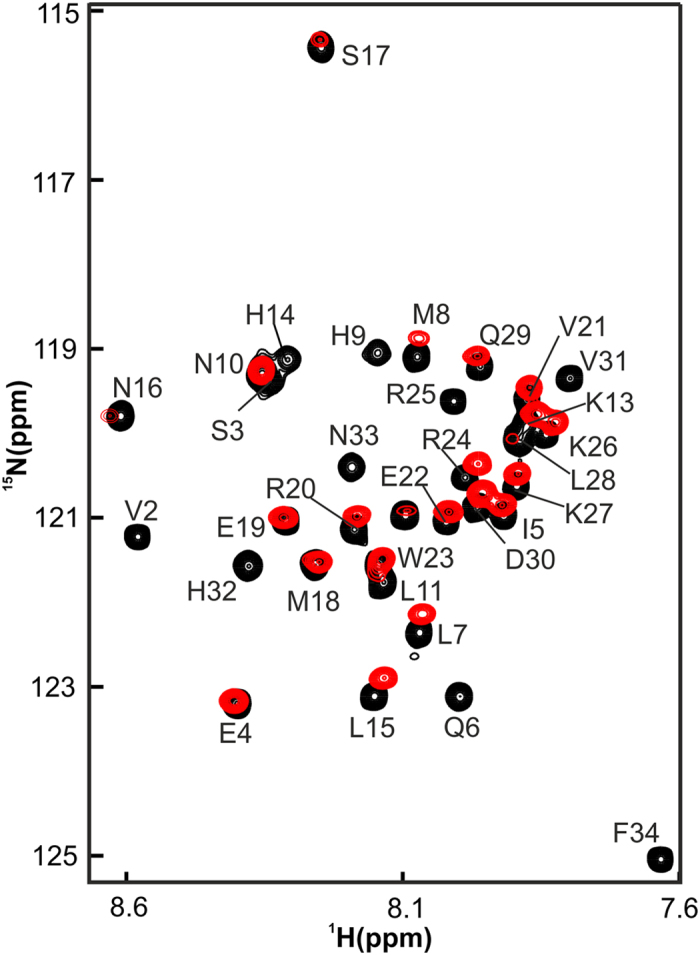
Binding of the Zn complex with PTH(1–34) mapped by NMR spectroscopy. Superimposed 2D ^1^H–^15^N HSQC spectra of ^15^N PTH(1–34) in the free state (black) and bound to the Zn complex (red).

NMR spectroscopy is a well suited method to investigate the exact binding sites, and conformational and/or dynamic changes upon protein–ligand interactions in solution^24^,^45^,^46^. Therefore, NMR titration experiments of ^15^N–PTH(1–34) and ^15^N–PTH(1–84) with the Zn complex were performed. The recorded ^15^N–HSQC experiment detects only backbone amides of ^15^N–PTH(1–34) or ^15^N–PTH(1–84) and suppresses all resonances of the Zn complex. A comparison of the spectra of ^15^N–PTH (1–34) in the free (black) and bound state (red) (Fig. 4) revealed differences in the chemical shifts and the line width of the resonances of individual residues. Many of the resonances were broadened beyond detection limits, possibly because of medium exchange rates on the NMR chemical shift time scale between free and bound PTH. Other residues change their chemical shift values. It is interesting to note that almost all residues showed changes in cross peak intensity and/or in chemical shift. This signifies that the whole peptide sequence of PTH(1–34) is involved in binding with the Zn complex.

**Figure 5:**
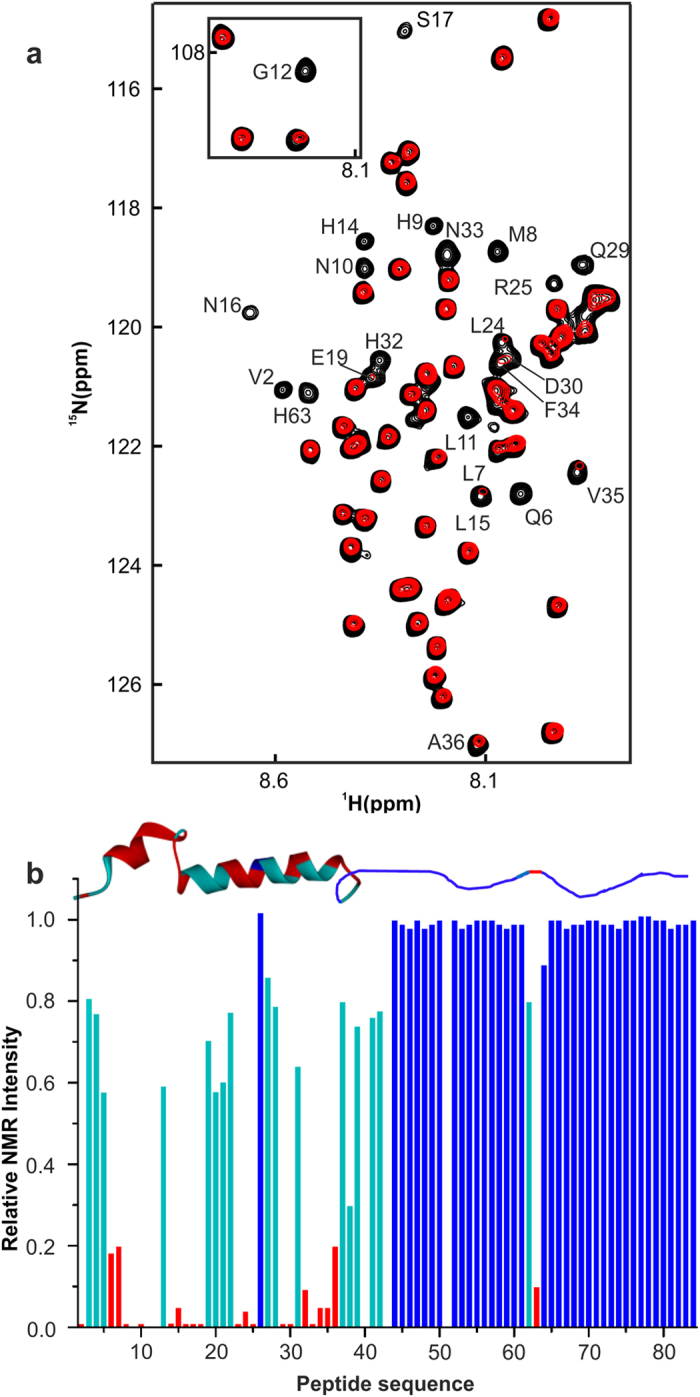
Binding profile of the Zn complex toward the PTH(1–84). (**a**) 2D ^1^H–^15^N HSQC overlaid spectrum of ^15^N PTH(1–84) in the free state (black) and bound to the Zn complex (red). (**b**) Residue by residue relative NMR intensity plot of ^15^N PTH(1–84) in the bound state with Zn complex compared to free ^15^N PTH(1–84). Residues lost >80% of intensity are marked red, 50–20% losses are given in cyan and no significant change in blue. The structure of PTH(1–84) (structure of residues 1–39, PDB: 1BWX)^63^ is displayed above the intensity plot. No bars are given for the prolines at positions 40, 43 and 51.

Preferential binding at residue-level resolution was further investigated by choosing full length ^15^N PTH(1–84) to see whether or not the C–terminal region is involved in the Zn complex interaction. A gross analysis showed that the Zn complex binds to N–terminal residues (Fig. 5a). At the end of the titration, several residues showed decreased NMR intensities. Notably, native resonances of residues V2, Q6, M8, H9, N10, L11, G12, H14, L15, N16, E19, W23, L24, R25, Q29, D30, H32, N33, F34, V35 and H63 were almost completely missing upon Zn complex addition. As a control, addition of 200 μM of Zn–perchlorate to PTH(1–84) did not show a significant change in NMR cross peaks (Figure S5). These data were quantified by comparing the intensity of each of the individual cross peaks of the PTH(1–84)/Zn complex with PTH(1–84) in the absence of the Zn complex (Fig. 5b). These NMR spectra indicate that the N–terminal domain of PTH (1–84) binds to the Zn complex whereas the C–terminus part remains solvent exposed and flexible enough to result in observable resonances. It is interesting to note that the aromatic residues (W23, F34) and all histidines (H9, H14, H32 and H63) plus their hydrophobic neighbouring residues are the hotspots of NMR intensity loss upon Zn complex addition, suggesting that their side chains facilitate binding. Histidine 63 is the only exception in the C–terminal part of PTH(1–84) sensing complex formation. In a control NMR titration experiment the interaction of 9-anthracenemethanol with PTH(1–84) showed no residue specific NMR intensity loss and thus the signature of an unspecific hydrophobic PTH peptide interaction (Figure S6).

The NMR data clearly demonstrated that the Zn complex binds preferably at the N–terminal domain of the peptide hormone irrespective of the peptide length. This possibly explained the binding behaviour observed by fluorescence spectroscopy and ITC which yielded nearly identical affinity. The additional Zn complex interactions of V35–A42 and S62–H63 in PTH(1–84) compared to PTH(1–34) have no significant contribution to the observed *K*_D_ values.

### Inhibition of cAMP pathway

**Figure 6:**
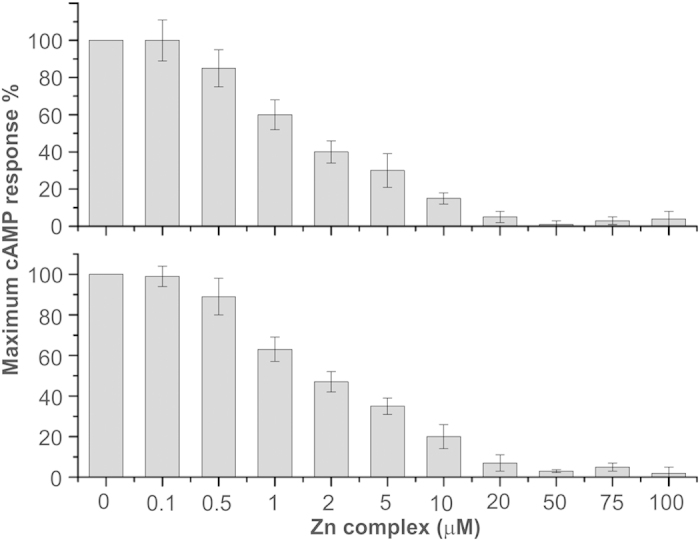
Inhibition of cAMP response by the Zn complex in stable HEK 293 cells expressing PTH1R. The Zn complex was incubated with peptide hormones prior to addition of cells. The upper panel shows the response from PTH(1–34) and the bottom panel for PTH(1–84). Error bars present the mean of three independent experimental runs in triplicate.

Fluorescence spectroscopy and ITC revealed the binding of the Zn complex with PTH(1–34) and PTH(1–84). NMR spectroscopy identified that the Zn complex interacts with the N–terminal part of the peptide hormone. The binding region from residues 1–37 of PTH(84) is important for recognition, interaction with the extracellular loops and trans–membrane helices of the receptor and bringing about activation of the later. There are two principal signal transduction pathways involving the G protein–coupled receptors: the cAMP signal pathway and the phosphatidylinositol signal pathway^47^. Parathyroid hormone is involved in the cAMP pathway for its biological regulation. Because binding of the Zn complex was at the N–terminal part of PTH, it is interesting to investigate the cAMP response towards its receptor in the presence of the Zn complex. Therefore PTH(1–34) and PTH(1–84) induced cAMP accumulation was measured in stable HEK 293 cell lines expressing recombinant PTH1R^33^. A cAMP generation assay was carried out in a Zn complex concentration dependent manner, keeping the cells and peptide hormone concentrations constant. PTH response without Zn complex was considered as 100% cAMP response. The peptide hormones were exposed to Zn complex for 15 minutes to allow for complex formation and subsequently cAMP generation was studied. A 50% reduced activity was observed at 2 μM of Zn complex and complete suppression of the cAMP response was observed at ~20 μM for both the peptides (Fig. 6). These results confirm that Zn complex binding at the N–terminal domain led to inactivation of the peptide hormones.

The cAMP generation was also analyzed to see the effect of the Zn complex on the receptor itself. Here, the Zn complex was pre-incubated with the cells followed by addition of PTH(1–84). This experiment did not show a significant suppression of cAMP generation inside the cells (black bars in Figure S7). The direct interaction of the Zn complex with the isolated N-terminus extracellular domain of PTH receptor 1 (nECD) was further analyzed by fluorescence spectroscopy where it showed a significantly weak affinity (*K*_D_ = 3.05 ± 0.2 mM) (Figure S8). In another control experiment cAMP generation was also studied by incubating the 9-anthracenemethanol with the peptide (Figure S9) or directly with the cells (grey bars in Figure S7). In both cases a significant suppression of intracellular cAMP generation was not observed. Additionally, inhibition of cAMP response or cell toxicity was also not observed up to 100 μM of Zn–perchlorate (Figure S10).

## Discussion

Here we are presenting a detailed analysis of the interaction of the Zn(II) complex of anthracenyl terpyridine with the human peptide hormones PTH(1–34) and PTH(1–84) and its modulating response towards their PTH1 receptors. Fluorescence and ITC studies suggested that at least two molecules of this Zn complex bind at the N–terminal domain of PTH. Binding at multiple sites is a common phenomenon for small ligands interacting with targeted proteins. For example, NMR spectroscopic analysis showed that two c–Myc–Max complex inhibitors, 10058–F4 and 10074–G5, bound to multiple regions of the monomeric c–Myc bHLHZip domain to inhibit the interaction between the binding partners^48^. Other examples are binding of epigallochatechin gallate (EGCG) to polypeptides, including α–synuclein and amyloid A*β* thereby redirecting the systems to nontoxic protein aggregates^49^,^50^ or EGCG inhibition of PTH(1–84) fibril growth^46^. Here the naturally secreted mature PTH peptide contains 84 residues, PTH(1–84), which is composed of three distinct regions: residues ~1–15 are involved in interaction with trans–membrane helices and activation of the receptor^51^,^52^, ~15–37 are responsible for receptor recognition at its ectodomain^53^,^54^,^55^, while the function of residues ~37–84 is not clear^56^. Both the free PTH peptide and the receptor bound state show α–helical secondary structure for residues ~1 to 34 (respective PDB codes are 1ZWB^39^ and 3C4M)^40^. The presented NMR data resulted in detected chemical shift changes of residues between 2 and ~42. The structured elements thus played an important role in interaction with the Zn complex.

All major NMR intensity losses of PTH(1–84) residues upon binding of the Zn complex occur close to the N-terminal aromatic and histidine residues of the peptide. Titration with Zn-perchlorate showed that free Zn(II) is unable to bind to PTH, thus highlighting that the organic skeletal system plays an important role in interaction. We further suggest that this system provides the specific hydrophobic interactions of PTH recognition because binding studies with 9-anthracenemethanol showed no preferential binding in the NMR titration experiment and only very weak affinity revealed by the fluorescence and ITC experiments.

The NMR detected chemical shift changes were observed at the N–terminal domain of the peptide hormone, thereby resulting in a suppressed cAMP response (Fig. 6). The decrease response was also detected by substitution of PTH residues. The tenth and eleventh residues of parathyroid hormone PTH(1–12) are important for stabilizing its helical conformation. The substitution of Leu7 with Phe7, Ala10 with Glu10 and/or the Arg11 with Ile11, markedly decreased cAMP generation^25^. It was also reported that the first, second, fourth, fifth, seventh, and eighth residue of PTH as well as W23 and F34, are important components of receptor binding/activation^53^,^57^. In the present studies these residues showed significant chemical shift changes and are thus expected to have an effect on the biological response.

The interaction of small molecules with proteins often occurs along the whole molecule e.g., NMR detected binding of EGCG to α–synuclein^49^, whilst here, the studied Zn complex showed the preferential binding at the N–terminal domain of the peptide hormones. Few synthetic molecules for example, 1,3,4–benzotriazepine non–peptide^58^ or peptide based DPC–AJ1951^59^ have been identified as parathyroid hormone–1 receptor (PTH1R) antagonists. These synthetic molecules act as activator for the receptor, while the present Zn complex acts as the inhibitor of an agonist. Mono– and bicyclic analogs of parathyroid hormone–related protein have been prepared and structurally characterized in a TFE:water mixture. However, their *in vitro* characterization (such as the generation of cAMP) has not been studied^60^,^61^.

A possible application of further developing such small molecules could be for combined treatments. In certain disease cases, such as hyperparathyroidism, it is necessary to control the function of PTH at the ligand level. So far, various treatments are available, e.g. by calcimimetics, bisphosphonates or hormone replacement therapy of parathyroid gland, to suppress the hormone secretion. However a continuous treatment has to be avoided due to hypercalcemia and hypercalciuria being frequently observed side effects^32^. Therefore, a combination of controlling the PTH response at the agonist level as presented here, along with suppression of PTH secretion, could be advantageous for immediate response and to improve therapeutic applications.

## Methods

### Protein expression and purification

Human PTH(1–34) and PTH(1–84) were purified as previously reported^23^. The expression plasmids for both the peptide hormones are based on a pET SUMO adapt vector containing C–terminal His–tags. Plasmids were transformed into *E. coli* BL21 codon+ cells. Uniform ^15^N isotope labeling of peptide hormones was achieved using M9 minimal medium supplemented with ^15^N NH_4_Cl. Peptide hormones were purified from soluble fractions using Ni–NTA affinity chromatography. Fractions containing the peptides were cleaved with SUMO protease (1:100 ratio) 50–150 μg/ml^62^. The cleaved fractions were further purified by S–75 gel filtration chromatography.

### Fluorescence spectroscopy

Fluorescence titrations were carried out on a JASCO FP6500. The samples were excited at 280 nm wavelength and the emission spectra were recorded in the range between 290 nm to 450 nm. Experiments were carried out in 10 mM BisTris, 300 mM Na_2_SO_4_, 0.02% NaN_3_, pH 7.2 using a 1 cm path length quartz cell. The concentration of PTH(1–34), PTH(1–84) and nECD-PTH1R used in the titration experiments were 7.4 μM, 8.1 μM and 5 μM, respectively. In a typical titration experiment, 4 μM of Zn complex or 9-anthracenemethanol was added and spectra were recorded after each addition. Zn complex or 9-anthracenemethanol in buffer was used as blank titration and these spectral traces were subtracted from the main titration. The Zn complex or 9-anthracenemethanol was dissolved in dimethyl sulfoxide and further diluted for experiments. The final concentration of DMSO was <1% in any experiment.

Fluorescence data were analyzed according to the following equation:


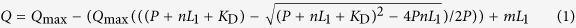


with *P* = (*P*_0_ / (*V*_0_ + *L*))*V*_0_ and *L*_1_ = (*L*_0_ / (*V*_0_ + *L*))*L* and *Q* – fluorescence intensity, *P* – protein concentration, *L* – ligand concentration, *V* – volume, *n* – binding sites and 0 – indicates start point.

### CD spectroscopy

CD spectra were recorded at 25 °C on a Jasco–J–810 spectropolarimeter with a scan speed of 20 nm/min. The measurements were carried out in 5 mM BisTris buffer at pH 7.2 with 15 μM of peptide. Titration experiments were carried out by addition of 10 μM of Zn complex at each time. Each spectrum reported is an average of four successive scans. The blank scans under same conditions were subtracted from the main spectral data.

### ITC experiment

The calorimetric titrations were performed at 25 °C with a MicroCal VPITC isothermal titration calorimeter. Small aliquots (7 μl) of the Zn complex were added to a solution of the respective peptide in the calorimeter cell. Successive additions were separated by a 300 second interval to allow the resulting peak from peptide Zn complex interaction to return to the baseline. All of the studies were carried out in 10 mM BisTris, 300 mM Na_2_SO_4_, pH 7.2.

### NMR spectroscopy

The spectra were recorded at the concentration of 50 μM of peptide hormones. Subsequently, titration was carried out with the addition of 0.5 μl of 10 mM Zn complex until there was no further change in NMR intensity observed. The spectrum corresponding to the 1:4 complex formations was used for the analysis. For the 9-anthracenemethanol titration experiment 20 μM of PTH was used. A titration was carried out and the 1:4 complex of PTH:9-anthracenemethanol was analyzed in detail. The spectra were corrected for the dilution factor. The previously determined NMR assignments of PTH resonances were used^24^,^63^,^64^. The spectra were recorded in the 10 mM BisTris, 300 mM Na_2_SO_4_, 0.02% NaN_3_, pH 5.3, with a Bruker 800 MHz Avance III spectrometer equipped with a CP–TCI cryoprobe at 25 °C. Spectra were processed using the programs NMRPipe and NMR Draw.

### cAMP accumulation assay

HEK 293 cells stably expressing hPTH1R were used for the cAMP accumulation assay following the previously reported procedure^24^,^33^. The reaction was carried out at 37 °C and fluorescence measurements were taken using a VICTOR X4 2030 plate reader (PerkinElmer Life and Analytical Sciences). A fixed amount of 0.01 μM peptide hormones and 4 × 10^3^ cells per well were used to monitor the cAMP response. The Zn complex or 9-anthracenemethanol was diluted form 0.1 μM to 100 μM and incubated for 15 minutes with the peptide hormone before addition of HEK 293 cells. All experiments were performed in triplicate and repeated independently three times. The maximum cAMP response were calculated as a percentage of the maximal response of PTH(1–34) or PTH(1–84). The effect of Zn complex or 9-anthracenemethanol on receptor was evaluated by incubating these with the cells for 15 minutes followed by washing with buffer. These treated cells were added to the reaction mixture containing untreated PTH(1–84) as described above.

## Additional Information

**How to cite this article**: Kumar, A. *et al*. Small Molecule Inhibited Parathyroid Hormone Mediated cAMP Response by N–Terminal Peptide Binding. *Sci. Rep*. **6**, 22533; doi: 10.1038/srep22533 (2016).

## Supplementary Material

Supplementary Information

## Figures and Tables

**Figure 1:**
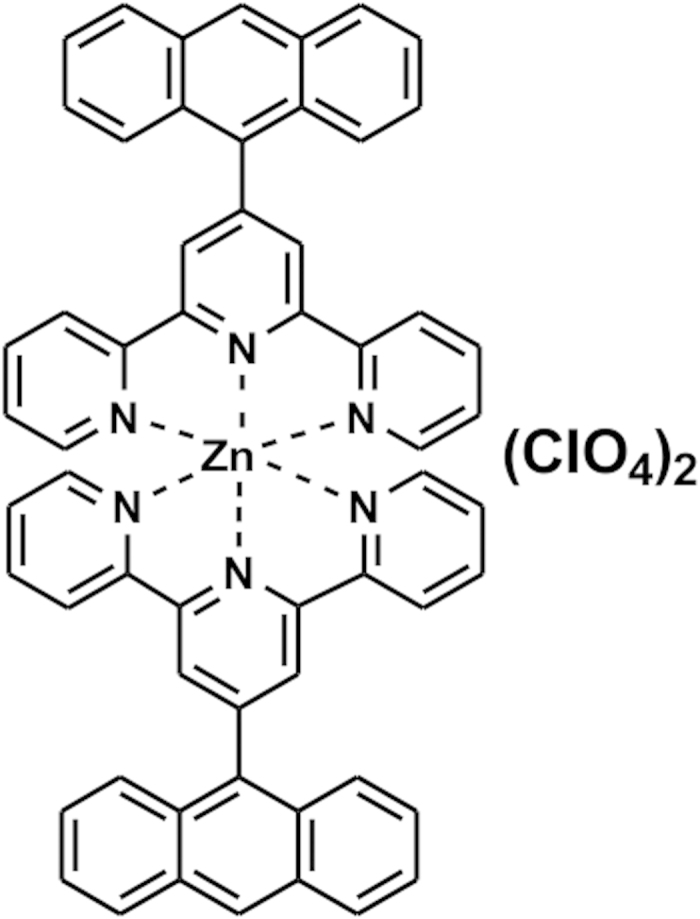
Line representation of the Zn^2+^ complex of anthracenyl terpyridine^9^.
