# Supplementary material for: Small Molecule Inhibited Parathyroid Hormone Mediated cAMP Response by N–Terminal Peptide Binding
Source: Sci Rep. 2016 Mar 2;6:22533. doi: 10.1038/srep22533 (PMC4773758; doi:10.1038/srep22533)
Supplement: Supplementary Information [file srep22533-s1.doc]

**Supporting information**

**Small Molecule Inhibited Parathyroid Hormone Mediated cAMP Response by N–Terminal Peptide Binding**

*Amit Kumar^1,2*^, Monika Baumann^2^ and Jochen Balbach^2,3*^*

**Figure S1**: Fluorescence intensity plots of poly peptide upon titration. (a) PTH(1–34) titrated with Zn complex, (b) PTH(1–84) titrated with Zn complex and (c) spectrum of PTH(1–84) and after addition of 200 μM of Zn–perchlorate.

**Figure S2**: Fluorescence intensity plots of PTH(1-84) upon titration with 9-anthracenemethanol. (a) Fluorescence spectra and (b) relative intensity plot indicating a *K*_D_ of 3.52 ± 0.38 mM.

**Figure S3**: CD spectral changes in PTH(1–84) upon titration of Zn complex. Black spectrum corresponds to free PTH(1–34). Spectrum beyond 215 nm could not be recorded due to the high absorption upon addition of Zn complex

**Figure S4**: ITC profile and best fit of the interaction of Zn complex with PTH(1–84). Top: raw data obtained from injections Zn complex into the PTH(1–84). Bottom represents the integrated curve showing the experimental points (■) and best fit (–).

**Figure S5**: Interaction of Zn–perchlorate and PTH(1–84). ^1^H–^15^N HSQC spectrum of PTH(1–84) before (black) and after (red) addition of 200 μM of Zn–perchlorate.


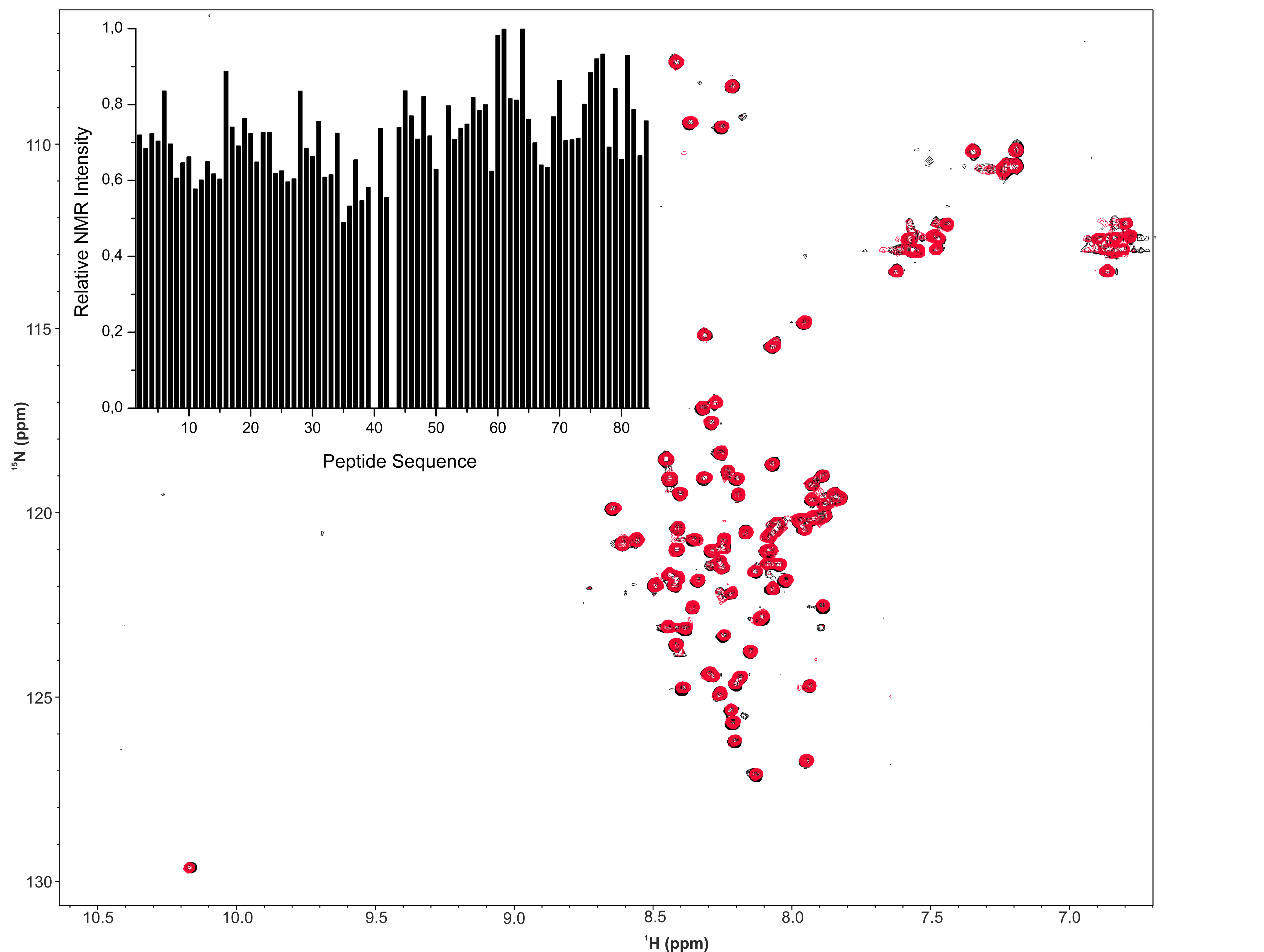


**Figure S6**: Interaction of 9-anthracenemethanol and PTH(1–84). (a) ^1^H–^15^N HSQC spectrum of PTH(1–84) before (black) and after (red) addition of 80 μM of 9-anthracenemethanol. (b) Relative intensity plots after addition of 4-fold access of 9-anthracenemethanol.

**Figure S7**: cAMP assay to see the effect on PTH1R by pre-incubating the inhibitor with cells followed by addition of agonist (PTH(1-84)). Black bars for Zn complex and grey bars for 9-anthracenemethanol.

**Figure S8**: Fluorescence binding of Zn complex with isolated N-terminus extracellular domain of PTH1R (nECD). (a) Fluorescence spectra and (b) relative intensity plot indicating the *K_d_* of 3.08 ± 0.2 mM.

**Figure S9**: cAMP assay in the presence of 9-anthracenemethanol. The experiment was carried out by pre-incubating the 9-anthracenemethanol with the peptide hormone (PTH(1-84)) followed by addition of cells.

**Figure S10**: (a) cAMP response and (b) cell viability against Zn–perchlorate. Cell viability was tested after 3h using MTT assay.
